# Average crop yield (2001–2017) in Ethiopia: Trends at national, regional and zonal levels

**DOI:** 10.1016/j.dib.2017.12.039

**Published:** 2017-12-19

**Authors:** Logan Cochrane, Yeshtila W. Bekele

**Affiliations:** a International and Global Studies, Carleton University, 2403R Richcraft Hall, Canada; b Center for Policy and Development Research, Hawassa University, Ethiopia

**Keywords:** Ethiopia, Agriculture, Yield data, Trends, Accuracy, Quality

## Abstract

This article presents average agricultural yield data per hectare for key cereal, legume and root crops from 2001 until 2017. Data was obtained from the annual Agricultural Sample Surveys of the Central Statistics Agency (CSA) of Ethiopia. We present data at national, regional (SNNPRS) and zonal (Wolaita) levels. The data shows that average yields for all crops, at all levels, show increasing trends during the time period. Data for the main cereal crops is consistent and aligns with literature relatively well, however we raise questions about the root crop data in an effort to encourage greater critical reflection of components of data from the CSA.

**Specification Table**

**Table:** 

Subject area	*Agriculture*
More specific subject area	*Crop yield data*
Type of data	*Figures and tables*
How data was acquired	*Data were obtained from the annual Agricultural Sample Surveys of the Central Statistics Agency of Ethiopia.*
Data format	*Analyzed*
Experimental factors	*Data used in this article were obtained from the Central Statistics Agency of Ethiopia, with reference to available literature.*
Experimental features	*Tables and graphic trends of analysis were employed.*
Data source location	*Ethiopia*
Data accessibility	*The data are with this article.*

**Value of the data**

• Average agricultural data are presented for key cereal, legume and root crops from 2001 to 2017.
• The data can be used by researchers and policy makers to analyze the implications of agriculture products on food security and poverty reduction.
• Average yields for all crops, at all levels, show increasing trends, with cereals doing so progressively and root crops increasing rapidly in recent years.
• Based upon some components of the governmental data, questions are raised about accuracy, encouraging researchers to be more critical when utilizing these data sets.

## 1 Data

**Fig. 1:**
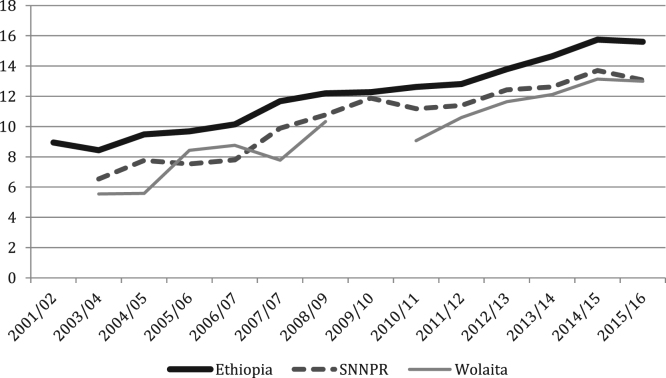
National, Regional and Zonal Average Teff Yield (Qt per Ha).

**Fig. 2:**
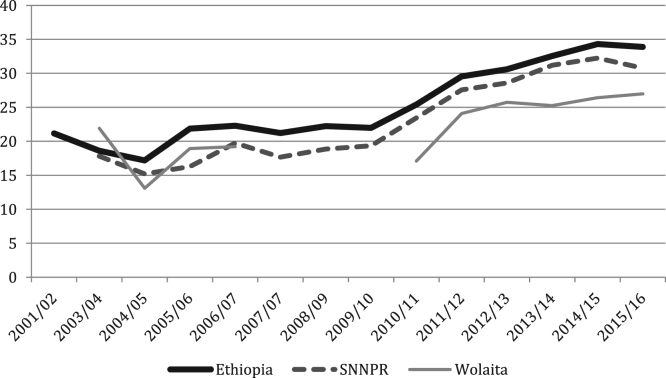
National, Regional and Zonal Average Maize Yield (Qt per Ha).

**Fig. 3:**
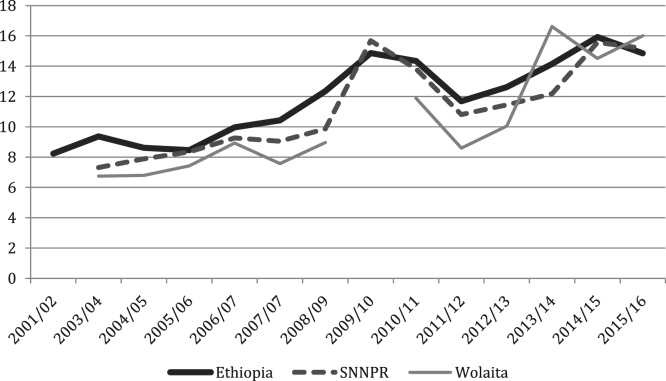
National, Regional and Zonal Average Haricot Bean (Qt per Ha).

**Fig. 4:**
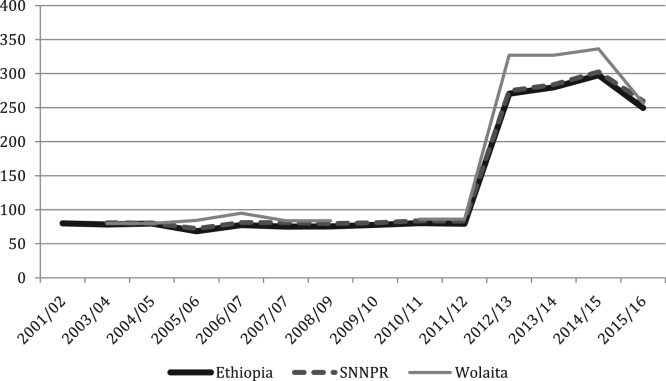
National, Regional and Zonal Average Taro Yield (Qt per Ha).

**Fig. 5:**
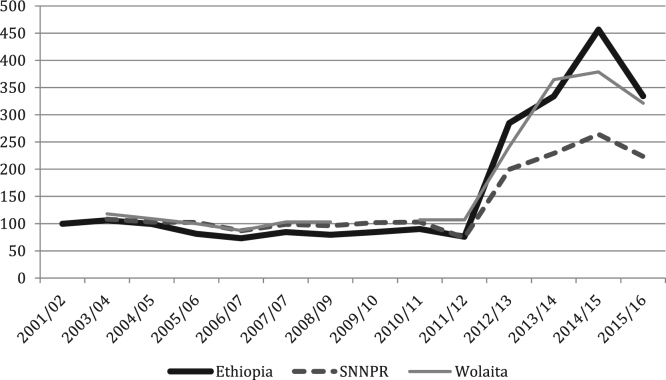
National, Regional and Zonal Average Sweet Potato Yield (Qt per Ha).

The figures and tables of agricultural data were obtained from the annual Agricultural Sample Surveys of the Central Statistics Agency (CSA) [1], [2], [3], [4], [5], [6], [7], [8], [9], [10], [11], [12], [13], covering the time period of 2001 until 2017. The CSA is the only provider of data at this scale. Average yields for all crops, at all levels, show increasing trends, with cereals doing so progressively and root crops increasing rapidly in recent years (Fig. 1, Fig. 2, Fig. 3, Fig. 4, Fig. 5). All the data is presented on a year-by-year basis in Table 1, Table 2, Table 3, enabling ease of re-analysis. However, there are general concerns about the quality, methodologies, and politicization of data produced by central statistics agencies [14]. We present data at national, regional (Southern Nations, Nationalities and Peoples’ regional state; SNNPRS) and zonal (Wolaita) scales. The data for the major cereals (teff and maize) is relatively consistent with the literature, whereas the shifts as well as contrasts with the literature in root crops raise questions about components of the agricultural data. For example,

1) In the 2012/13 season yields per hectare of taro and sweet potato tripled, according to CSA personnel this was due to methodological changes (Tables 4–5) [15];
2) Data for sweet potato are stable in SNNPRS from 2007 to 2011, but during this period sweet potato virus infection was high, affecting roots, weights and cuttings yet no decline was recorded (Table 5) [16];
3) During the 2007/08 season a higher yielding variety of taro was introduced with high adoption rates but no increase was recorded in the years that followed (Table 4) [15];

**Table 1:** Ethiopia Yields by Crop (Qt per Ha).

Year	Crop	Yield
2001/02	Teff	8.95
2003/04	Teff	8.43
2004/05	Teff	9.48
2005/06	Teff	9.69
2006/07	Teff	10.14
2007/08	Teff	11.67
2008/09	Teff	12.2
2009/10	Teff	12.28
2010/11	Teff	12.62
2011/12	Teff	12.81
2012/13	Teff	13.79
2013/14	Teff	14.65
2014/15	Teff	15.75
2015/16	Teff	15.60
2001/02	Maize	21.16
2003/04	Maize	18.6
2004/05	Maize	17.19
2005/06	Maize	21.87
2006/07	Maize	22.29
2007/08	Maize	21.22
2008/09	Maize	22.24
2009/10	Maize	21.99
2010/11	Maize	25.4
2011/12	Maize	29.54
2012/13	Maize	30.59
2013/14	Maize	32.54
2014/15	Maize	34.31
2015/16	Maize	33.87
2001/02	Haricot Beans	8.23
2003/04	Haricot Beans	9.37
2004/05	Haricot Beans	8.61
2005/06	Haricot Beans	8.46
2006/07	Haricot Beans	9.97
2007/08	Haricot Beans	10.43
2008/09	Haricot Beans	12.35
2009/10	Haricot Beans	14.87
2010/11	Haricot Beans	14.34
2011/12	Haricot Beans	11.69
2012/13	Haricot Beans	12.62
2013/14	Haricot Beans	14.15^a^
2014/15	Haricot Beans	15.92^a^
2015/16	Haricot Beans	14.85^a^
2001/02	Taro	79.93
2003/04	Taro	78.39
2004/05	Taro	79.69
2005/06	Taro	68.50
2006/07	Taro	77.43
2007/08	Taro	75.29
2008/09	Taro	75.45
2009/10	Taro	77.78
2010/11	Taro	80.37
2011/12	Taro	79.41
2012/13	Taro	270.4
2013/14	Taro	279.8
2014/15	Taro	297.81
2015/16	Taro	249.61
2001/02	Sweet Potato	99.67
2003/04	Sweet Potato	105.91
2004/05	Sweet Potato	99.42
2005/06	Sweet Potato	81.40
2006/07	Sweet Potato	73.06
2007/08	Sweet Potato	84.43
2008/09	Sweet Potato	79.48
2009/10	Sweet Potato	84.31
2010/11	Sweet Potato	90.13
2011/12	Sweet Potato	76.03
2012/13	Sweet Potato	284.64
2013/14	Sweet Potato	334.04
2014/15	Sweet Potato	456.56
2015/16	Sweet Potato	334.39

a CSA began dividing white and red haricot beans in 2013/14, the figures used are an average of the two.

**Table 2:** SNNPRS Yields by Crop (Qt per Ha).

Year	Crop	Yield
2003/04	Teff	6.54
2004/05	Teff	7.76
2005/06	Teff	7.53
2006/07	Teff	7.80
2007/08	Teff	9.89
2008/09	Teff	10.77
2009/10	Teff	11.88
2010/11	Teff	11.18
2011/12	Teff	11.40
2012/13	Teff	12.43
2013/14	Teff	12.62
2014/15	Teff	13.7
2015/16	Teff	13.08
2003/04	Maize	17.8
2004/05	Maize	15.21
2005/06	Maize	16.27
2006/07	Maize	19.73
2007/08	Maize	17.67
2008/09	Maize	18.85
2009/10	Maize	19.33
2010/11	Maize	23.45
2011/12	Maize	27.57
2012/13	Maize	28.57
2013/14	Maize	31.18
2014/15	Maize	32.23
2015/16	Maize	30.75
2003/04	Haricot Beans	7.31
2004/05	Haricot Beans	7.89
2005/06	Haricot Beans	8.35
2006/07	Haricot Beans	9.26
2007/08	Haricot Beans	9.05
2008/09	Haricot Beans	9.87
2009/10	Haricot Beans	15.68
2010/11	Haricot Beans	13.82
2011/12	Haricot Beans	10.81
2012/13	Haricot Beans	11.44
2013/14	Haricot Beans	12.18^a^
2014/15	Haricot Beans	15.56^a^
2015/16	Haricot Beans	15.18^a^
2003/04	Taro	80.87
2004/05	Taro	80.40
2005/06	Taro	72.47
2006/07	Taro	81.03
2007/08	Taro	81.11
2008/09	Taro	79.77
2009/10	Taro	80.88
2010/11	Taro	83.65
2011/12	Taro	83.10
2012/13	Taro	274.84
2013/14	Taro	283.72
2014/15	Taro	302.61
2015/16	Taro	259.87
2003/04	Sweet Potato	108.2
2004/05	Sweet Potato	103.01
2005/06	Sweet Potato	102.12
2006/07	Sweet Potato	86.83
2007/08	Sweet Potato	98.72
2008/09	Sweet Potato	96.17
2009/10	Sweet Potato	101.78
2010/11	Sweet Potato	102.84
2011/12	Sweet Potato	74.08
2012/13	Sweet Potato	199.55
2013/14	Sweet Potato	228.92
2014/15	Sweet Potato	264.14
2015/16	Sweet Potato	223.19

a CSA began dividing white and red haricot beans in 2013/14, the figures used are an average of the two.

**Table 3:** Wolaita Yields by Crop (Qt per Ha).

Year	Crop	Yield
2003/04	Teff	5.54
2004/05	Teff	5.59
2005/06	Teff	8.43
2006/07	Teff	8.76
2007/08	Teff	7.78
2008/09	Teff	10.33
2009/10	Teff	–
2010/11	Teff	9.07
2011/12	Teff	10.60
2012/13	Teff	11.64
2013/14	Teff	12.11
2014/15	Teff	13.14
2015/16	Teff	13.00
2003/04	Maize	21.91
2004/05	Maize	13.08
2005/06	Maize	18.92
2006/07	Maize	19.21
2007/08	Maize	–
2008/09	Maize	18.19
2009/10	Maize	–
2010/11	Maize	17.09
2011/12	Maize	24.10
2012/13	Maize	25.73
2013/14	Maize	25.24
2014/15	Maize	26.42
2015/16	Maize	26.99
2003/04	Haricot Beans	6.75
2004/05	Haricot Beans	6.79
2005/06	Haricot Beans	7.42
2006/07	Haricot Beans	8.92
2007/08	Haricot Beans	7.57
2008/09	Haricot Beans	8.96
2009/10	Haricot Beans	–
2010/11	Haricot Beans	11.88
2011/12	Haricot Beans	8.60
2012/13	Haricot Beans	10.05
2013/14	Haricot Beans	16.62^a^
2014/15	Haricot Beans	14.51
2015/16	Haricot Beans	16.00
2003/04	Taro	80
2004/05	Taro	80.00
2005/06	Taro	84.40
2006/07	Taro	94.81
2007/08	Taro	83.84
2008/09	Taro	83.84
2009/10	Taro	–
2010/11	Taro	86.06
2011/12	Taro	86.06
2012/13	Taro	327
2013/14	Taro	327
2014/15	Taro	336.4
2015/16	Taro	256.4
2003/04	Sweet Potato	118
2004/05	Sweet Potato	109.00
2005/06	Sweet Potato	100.00
2006/07	Sweet Potato	87.53
2007/08	Sweet Potato	102.91
2008/09	Sweet Potato	102.91
2009/10	Sweet Potato	–
2010/11	Sweet Potato	106.79
2011/12	Sweet Potato	106.79
2012/13	Sweet Potato	241
2013/14	Sweet Potato	364.54
2014/15	Sweet Potato	378.66
2015/16	Sweet Potato	321.29

a CSA began dividing white and red haricot beans in 2013/14, the figures used are an average of the two.

Alternative surveys of the required scale do not appear feasible or realistic at this time. However, the questions above highlight the need for more research to assess the data provided by central statistics agencies. Often these data sets are utilized without critical reflection about quality, methodology or politicization.

## 2 Experimental design, materials and methods

**Map 1:**
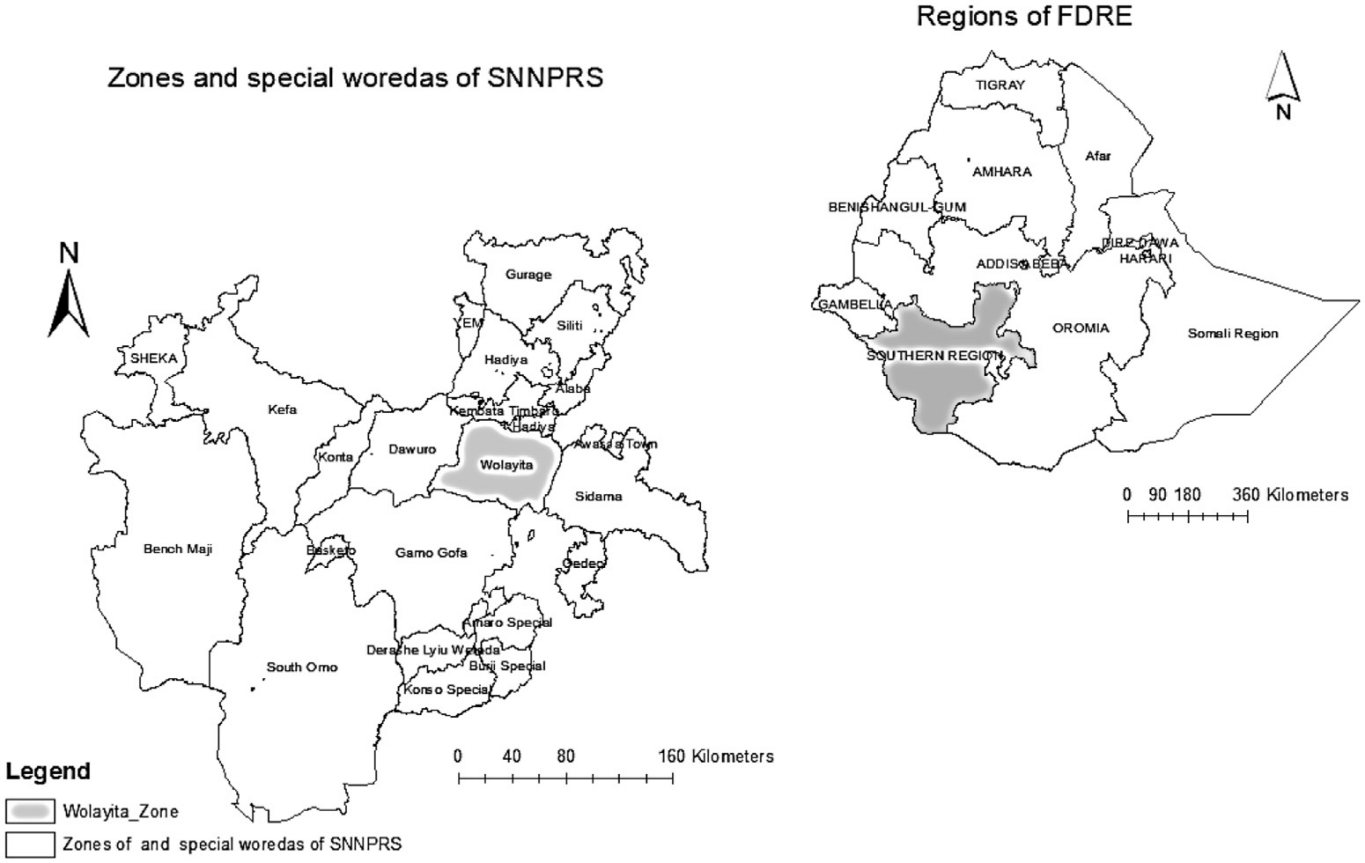
National (FDRE), Regional (SNNPRS) and Zonal (Wolaita).

Average crop yield data at national, regional (SNNPRS) and zonal (Wolaita) levels (see Map 1) were obtained from the CSA annual Agricultural Sample Surveys. The data is presented using figures to highlight trends and tables to allow for further analyses of the data. We have selected SNNPRS as an example region and Wolaita as an example zone primarily due to our familiarity with the areas respectively, and thus enhancing our ability to identify questions. The objective of raising questions about the agricultural root crop yield data is to encourage researchers to engage with central statistics data more critically. This does not suggest that the CSA data is inaccurate; rather it acts an encouragement for CSA data to be a subject of greater study.
