# Supplementary material for: Average crop yield (2001–2017) in Ethiopia: Trends at national, regional and zonal levels
Source: Data Brief. 2017 Dec 19;16:1025–33. doi: 10.1016/j.dib.2017.12.039 (PMC5758922; doi:10.1016/j.dib.2017.12.039)
Supplement: Supplementary file 1 — Transparency document [file mmc1.docx]

**Disclosure statements**

There is no potential conflict of interest reported by the authors.
